# Microorganism-Templated Nanoarchitectonics of Hollow TiO_2_-SiO_2_ Microspheres with Enhanced Photocatalytic Activity for Degradation of Methyl Orange

**DOI:** 10.3390/nano12091606

**Published:** 2022-05-09

**Authors:** Shenglan Liao, Liqin Lin, Jiale Huang, Xiaolian Jing, Shiping Chen, Qingbiao Li

**Affiliations:** 1 Department of Chemical and Biochemical Engineering, College of Chemistry and Chemical Engineering, Xiamen University, Xiamen 361005, China; shenglanliao@outlook.com (S.L.); cola@xmu.edu.cn (J.H.); kelqb@xmu.edu.cn (Q.L.); 2 Department of Chemical Engineering and Pharmacy, College of Chemical Engineering and Materials Science, Quanzhou Normal University, Quanzhou 362000, China; jingxiaolian@foxmail.com (X.J.); shiping22@163.com (S.C.)

**Keywords:** hollow SiO_2_ microspheres, biotemplate, TiO_2_-SiO_2_, photocatalyst

## Abstract

In this study, hollow SiO_2_ microspheres were synthesized by the hydrolysis of tetraethyl orthosilicate (TEOS) according to the Stober process, in which *Pichia pastoris* GS 115 cells were served as biological templates. The influence of the preprocessing method, the TEOS concentration, the ratio of water to ethanol, and the aging time on the morphology of microspheres was investigated and the optimal conditions were identified. Based on this, TiO_2_-SiO_2_ microspheres were prepared by the hydrothermal process. The structures and physicochemical properties of TiO_2_-SiO_2_ photocatalysts were systematically characterized and discussed. The photocatalytic activity for the degradation of methyl orange (MO) at room temperature under Xe arc lamp acting as simulated sunlight was explored. The result showed that the as-prepared TiO_2_-SiO_2_ microspheres exhibited a good photocatalytic performance.

## 1. Introduction

The rapid development of the textile industry not only brings considerable economic benefits, but also aggravates environmental pollution. Due to the complex composition of textile wastewater and its high content of organic substances, harmful substances, and deep chroma, which cause serious harm to water bodies, the treatment of textile wastewater is imperative [1,2]. Water-soluble azo dyes including MO are the main targets of pollution control. Many treatment methods of dye removal have been investigated, including adsorption [3,4], photo-Fenton oxidation [5,6,7], H_2_O_2_/UV (ultraviolet) treatment [8,9], photocatalysis [10,11], and biological treatment [12,13,14]. Among these methods, photocatalysis is considered an effective method to degrade dyes in wastewater [15].

TiO_2_ is considered to be one of the most promising photocatalysts for the removal of organic pollutants in textile wastewater due to its low cost, strong oxidizing properties, non-toxicity, and biochemical inertness [16,17,18]. However, there are some imperfections, such as its large energy gap, high photoelectric hole recombination rate, etc. [19,20,21,22]. The small nanoparticles have a high surface energy and readily form agglomerates, and their wide band gap (3.2 eV) makes them inactive under visible light irradiation. Moreover, the application in high temperature and high-pressure reactions is limited due to the poor mechanical strength and thermal stability of TiO_2_. Therefore, researchers are looking for more effective methods with which to improve the surface-active sites of TiO_2_, the photoelectron-hole separation rate, the solar energy usage efficiency, and the spectral response to enhance its photocatalytic performance [23]. One of the most promising methods to enhance the activity of TiO_2_ under visible light and sunlight irradiation is to dope non-metals such as N, C, and Si. Due to the difference in properties between Si and Ti, mesoporous SiO_2_ is more stable than mesoporous TiO_2_. Mesoporous TiO_2_ can easily cause the collapse of the mesoporous structure when the template is removed by calcination at high temperatures, while silicon-based materials have a good thermal stability. Taking advantage of the good thermal stability of silicon-based materials, titanium-silicon composites could be prepared by loading titanium onto mesoporous silicon-based materials, which can effectively solve the pore structure instability of mesoporous TiO_2_ [20].

**Figure A1:**
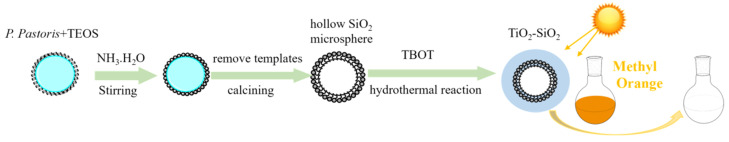
Reaction mechanism diagram.

The methods used for combining TiO_2_ and SiO_2_ can be roughly divided into two categories. One involves the mechanical mixture of TiO_2_ and SiO_2_. The other involves the use of chemical methods such as co-precipitation and sol–gel, where the composite oxide TiO_2_-SiO_2_ with Ti-O-Si bonds is obtained [24,25,26]. It is generally believed that TiO_2_-SiO_2_ oxides with Ti-O-Si bonds perform better as catalyst supports than mechanically mixed TiO_2_-SiO_2_ [27]. Among various composite oxides, TiO_2_-SiO_2_ composite oxides, especially in the mesoporous structure, exhibit a good chemical stability, availability, reusability, and controllability of pore structure [28]. Compared with other chemical methods, hydrothermal methods are often applied for the preparation of metal oxide materials because of their characteristics of a fast reaction speed, an adjustable structure, and crystallinity. In this study, microorganism cells-templated TiO_2_-SiO_2_ hollow microspheres were synthesized by the combination of the Stober process and the hydrothermal process and the photocatalytic activity for the degradation of methyl orange was investigated (see Figure A1).

## 2. Materials and Methods

### 2.1. Materials

TEOS (tetraethyl orthosilicate, purity ≥ 98%), TBOT (titanium butoxide, purity ≥ 99%), 25% ammonia solution (purity ≥ 99.5%), nitric acid (65.0~68.0%), methyl orange, and ethanol (purity ≥ 99.7%) were used without further purification. *Pichia pastoris* GS115 (*P. pastoris* GS115) cells were cultured in our laboratory.

### 2.2. Methods

#### 2.2.1. Preparation of Hollow SiO_2_ Microspheres

Hollow SiO_2_ microspheres were synthesized using microorganism cells as templates according to the Stober process. In a typical experiment, 0.2 g of *P. pastoris* GS115 cells were suspended in 4.2 mL of ultrapure water and 10.5 mL of absolute ethanol (the ratio of water–ethanol was 1/2). The mixture was placed on a magnetic stirrer to stir at 25 °C for 1 h. Then, 6.4 mL of TEOS (1.2 mol/L, if not specified) was added and allowed to react for 2 h, followed by 2.85 mL of ammonia for another 1 h. The suspension was agitated at 25 °C for 12 h (if not specified). The product was separated by centrifugation (3500 r/min, 10 mins) and washed with ethanol and water several times. The precipitate was dried and calcinated at 550 °C for 2 h with a heating rate of 2 °C/min.

#### 2.2.2. Preparation of TiO_2_-SiO_2_

A total of 2 g of the as-synthesized hollow SiO_2_ microspheres was dispersed in 25 mL of anhydrous ethanol and 0.25 mL of TBOT. The solution was labeled as solution A. Then, 0.2 mL of nitric acid was added into the mixture of anhydrous ethanol (25 mL) and ultra-pure water (10 mL). The solution was labeled as solution B. Solution A was continuously stirred at 25 °C for 5 min, and then solution B was added drop by drop and stirred for another 2 h. The mixture was transferred to a reaction kettle and heated at 180 °C for 24 h. After the hydrothermal reaction was completed, the samples were separated by centrifugation (3500 r/min, 10 min) and washed alternately with water and ethanol several times. The obtained samples were dried in an oven at 80 °C for 10 h and calcinated at 550 °C for 1 h with a heating rate of 2 °C/min.

#### 2.2.3. Determination of Photocatalytic Performance

The photocatalytic experiments were carried out in a glass vessel by a 300 W Xe arc lamp acting as simulated sunlight. The initial concentration of the methyl orange was 10 mg/L. A total of 80 mg of the photocatalyst was taken in 80 mL of MO solution. Illumination was implemented after dark treatment for 1 h to reach adsorption–desorption equilibrium. At specific time intervals (every 20 min), 4 mL of the sample was taken from the suspensions and centrifuged to remove the catalyst prior to spectral measurement.

### 2.3. Characterization Methods

The crystal structure of the samples was determined by an X-ray diffractometer (XRD, X’Pert Pro MPD, Panalytical, The Netherlands) operated at a voltage of 40 kV and a current of 30 mA with Cu Kα radiation. The observations of morphology and microstructure were performed on a scanning electron microscope (SEM, ZEISS Sigma, Oberkochen, Germany) and a transmission electron microscope (TEM, Philips Tecnai F30, Eindhoven, The Netherlands) operated at an accelerating voltage of 300 kV. The specific surface area, pore volume, and pore size distribution of the samples were determined by Tristar-type low-temperature N_2_ physical adsorption and desorption (BET, NOVA2200e, Quantachrome, Boynton Beach, FL, USA). The thermogravimetric (TG) studies were carried out on Netzsch TG209F1 thermobalance (NETZSCH Scientific Instruments Co., Selb, Germany) under a flowing-air atmosphere at a heating rate of 10 °C/min. X-ray Photoelectron Spectroscopy (XPS) measurements were performed on a PHI 5000 versa probe-II microprobe (Ulvac-Phi, Kanagawa, Japan). The UV-DRS analysis was performed on a UV-VIS Cary 5000 instrument (Varian, Palo Alto, CA, USA). BaSO_4_ was served as a reference. The Fourier Transform Infrared Spectroscopy (FTIR) analysis was carried out on a Nicolet 6700 instrument (Thermo Fisher Scientific, Waltham, MA, USA).

## 3. Results and Discussion

### 3.1. Preparation of Hollow SiO_2_ Microspheres

In recent years, inorganic hollow micro/nanostructures have attracted extensive attention due to their unique morphologies, unique physicochemical properties, and potential applications in dyes, drug delivery, and efficient catalysis [29,30]. The template method is one of the most commonly used methods for the synthesis of hollow nanomaterials [31]. The application of microorganisms as a template is considered to be an economical and green method [32,33,34,35]. Herein, the hollow SiO_2_ was prepared by using the *P. pastoris* GS115 cells as a template and the influence of different reaction conditions on the structure were investigated.

#### 3.1.1. Effect of Preprocessing Methods

**Figure 1:**
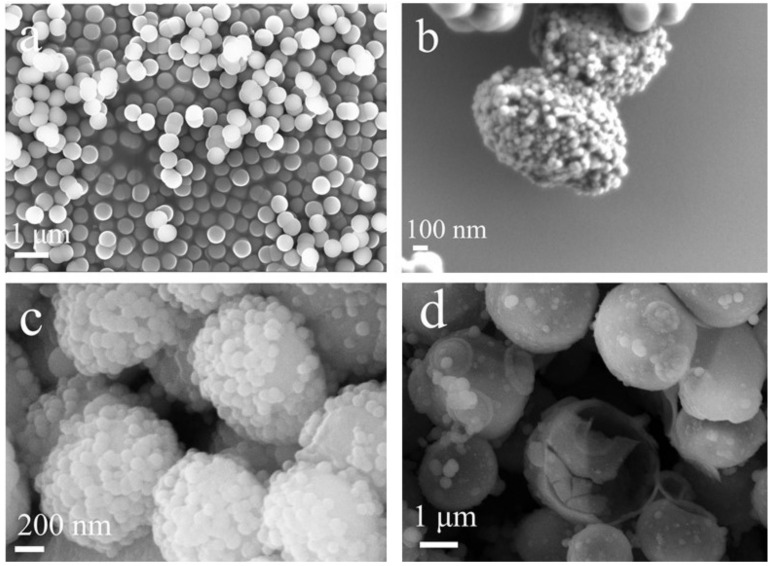
SEM images of SiO_2_ prepared by different preprocessing methods: (**a**) without adding template; (**b**) *P. pastoris* GS 115 were suspended in ethanol and followed by TEOS and ammonia; (**c**) *P. pastoris* GS 115 were suspended in a hybrid system of ethanol and ammonia and followed by TEOS and ammonia; (**d**) *P. pastoris* GS 115 were suspended in an ethanol–water mixture and followed by TEOS and ammonia.

Templates are vital for the preparation of hollow material. As shown in Figure 1a, solid SiO_2_ microspheres with a particle size of 200–300 nm were obtained when no template was introduced. When *P. pastoris* GS 115 cells with the size of 1–2 μm were introduced, the obtained microspheres had successfully replicated the template structure (Figure 1b–d). The solvent has an obvious influence on the morphology. When *P. pastoris* GS 115 cells were suspended in ethanol or a hybrid system of ethanol and ammonia, there were many nano SiO_2_ particles on the surface of hollow SiO_2_, making the surface more rough and still agglomerate, which was shown in Figure 1b,c. While hollow, SiO_2_ with a smooth surface and a good dispersion could be prepared if *P. pastoris* GS115 cells were firstly suspended in a water–ethanol mixture (the ratio of water–ethanol is 1/2, Figure 1d).

#### 3.1.2. Effect of TEOS Concentration

**Figure 2:**
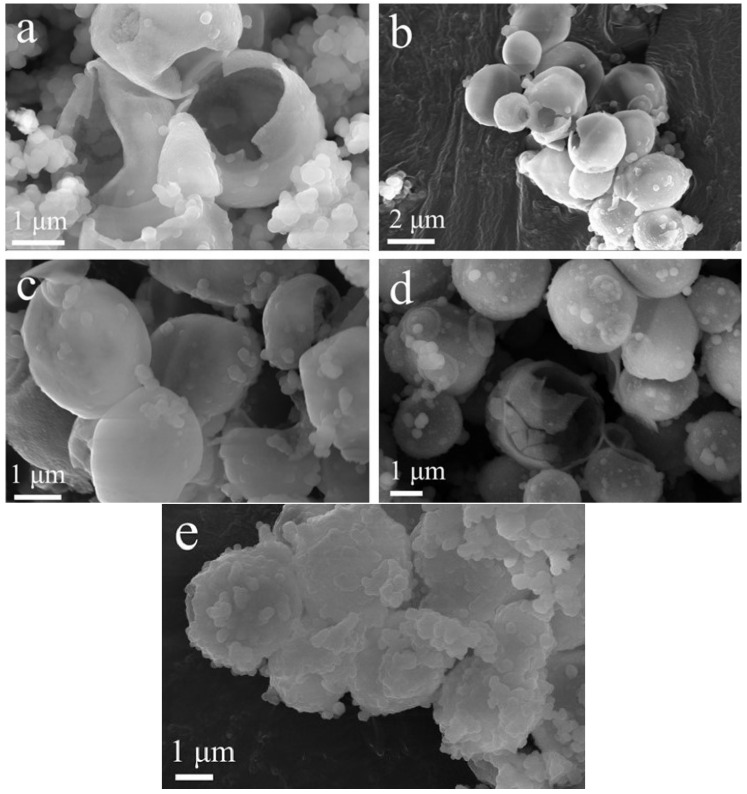
SEM images of SiO_2_ synthesized at different TEOS concentrations: (**a**) 0.9 mol/L; (**b**) 1.0 mol/L; (**c**) 1.1 mol/L; (**d**) 1.2 mol/L; (**e**) 1.3 mol/L.

The concentration of TEOS has an effect on the morphology of microspheres. When the TEOS concentration was low, small SiO_2_ particles agglomerated and the concentration was not enough to form the complete hollow structure, as shown in Figure 2a. As the TEOS concentration gradually increased, more complete microspheres with hollow structure could successfully be prepared (Figure 2b–d). As the concentration of TEOS continued to increase, the excess SiO_2_ particles continued to grow on the surface of the hollow SiO_2_ microspheres due to the limited amount of templates. The surface of the prepared microspheres was relatively rough and agglomerated together to form larger clusters (Figure 2e). Therefore, to prepare hollow SiO_2_ microspheres with a smooth surface and good dispersibility, the optimal TEOS concentration is between 1.0 and 1.2 mol/L.

#### 3.1.3. Effect of the Ratio of Water to Ethanol

**Figure 3:**
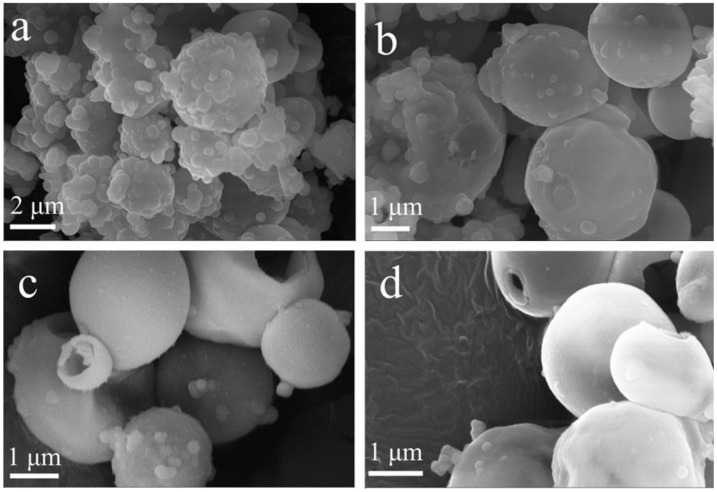
SEM images of SiO_2_ synthesized at different ratios of water to ethanol: (**a**) 1/1; (**b**) 1/1.5; (**c**) 1/2; (**d**) 1/2.5.

As shown in Figure 3, when the ratio of water to ethanol was low, the generated SiO_2_ particles would continue to grow on the surface of hollow SiO_2_ microspheres, which made the surface of the prepared microspheres relatively rough and caused the hollow microspheres to have a certain degree of agglomeration (Figure 3a). With the increase in water/ethanol, the surface of the microspheres became smoother (Figure 3b–d).

#### 3.1.4. Effect of Aging Time

**Figure 4:**
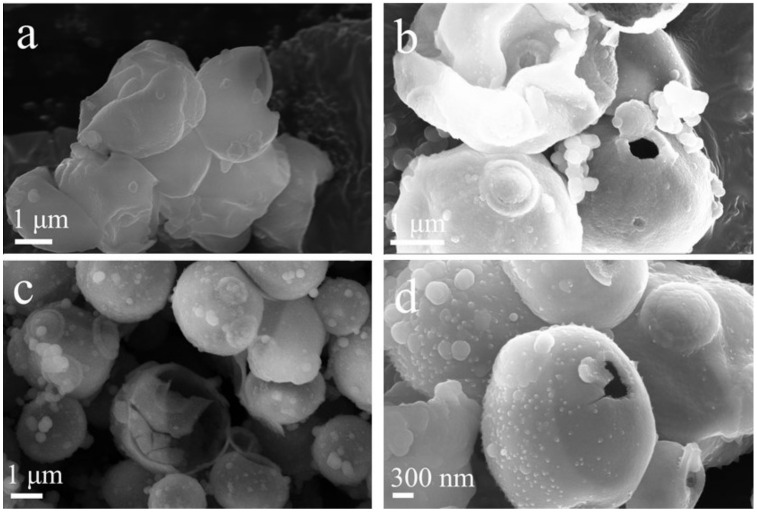

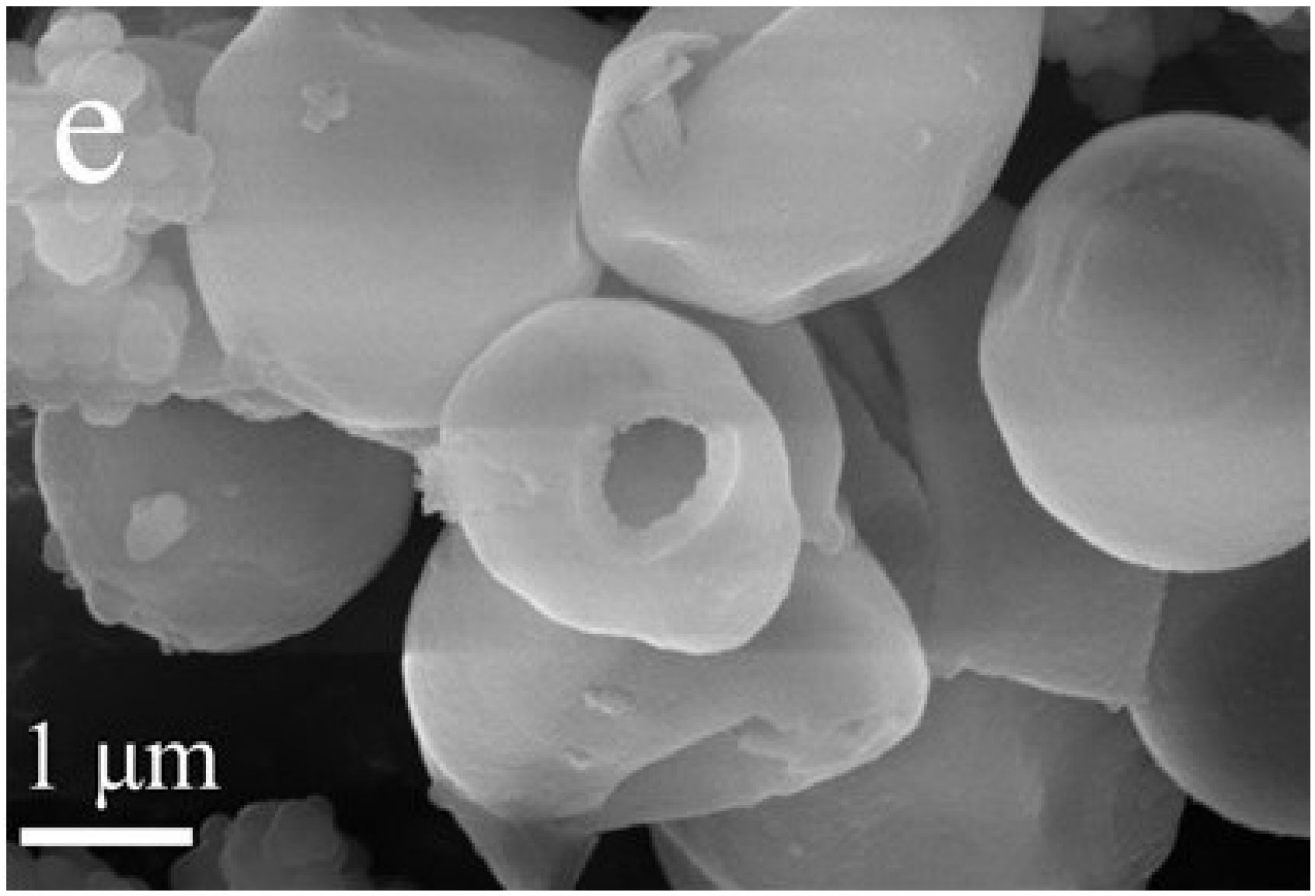
SEM images of SiO_2_ synthesized at different aging times: (**a**) 6 h; (**b**) 10 h; (**c**) 12 h; (**d**) 24 h; (**e**) 36 h.

The aging time also has an obvious effect on the surface of microspheres. As shown in Figure 4a, when the aging time was 6 h, the hollow SiO_2_ microsphere structure was irregular and the dispersion was poor. With the aging time extended to 12 h, a complete hollow SiO_2_ microsphere structure was formed with an even dispersion (Figure 4c). Complete hollow microsphere structures could be formed by extending the aging time (Figure 4d,e).

**Figure A2:**
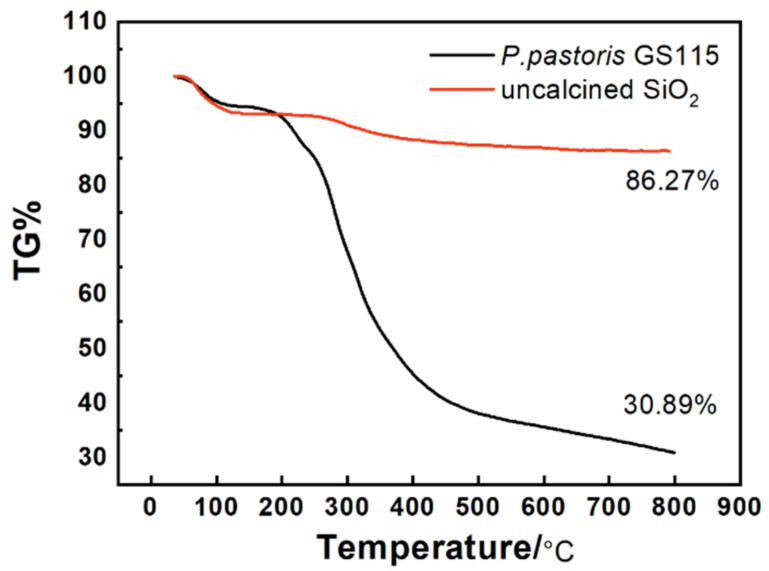
TG curves of *P. pastoris* GS115 and uncalcined SiO_2_ (conditions: TEOS concentration 1.2 mol/L, the ratio of water to ethanol 1/2, aging time 12 h).

**Figure A3:**
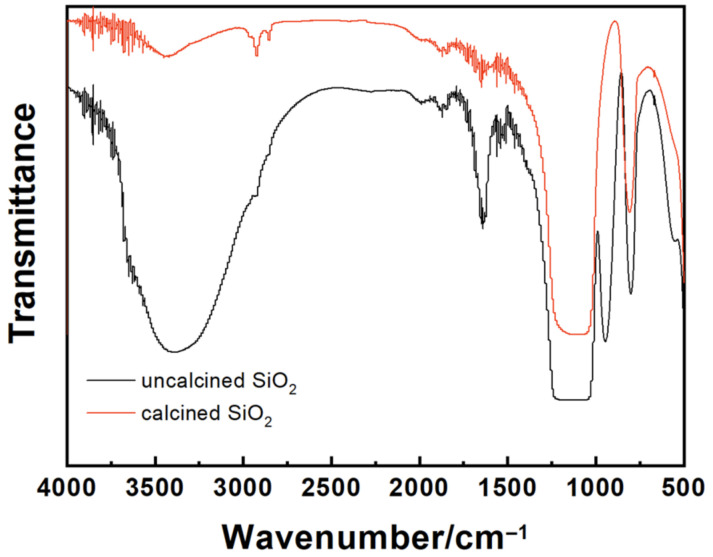
FT-IR spectra of SiO_2_.

The TG and FTIR characterizations were performed and the results were shown in Figure A2 and Figure A3. The results confirm the formation of SiO_2_ and show that there may be some residual biomass on the microsphere.

### 3.2. Preparation of Hollow TiO_2_-SiO_2_

Based on the above, TiO_2_ was coated on the surface of hollow SiO_2_ microspheres to prepare TiO_2_-SiO_2_ by the hydrothermal method.

**Figure 5:**
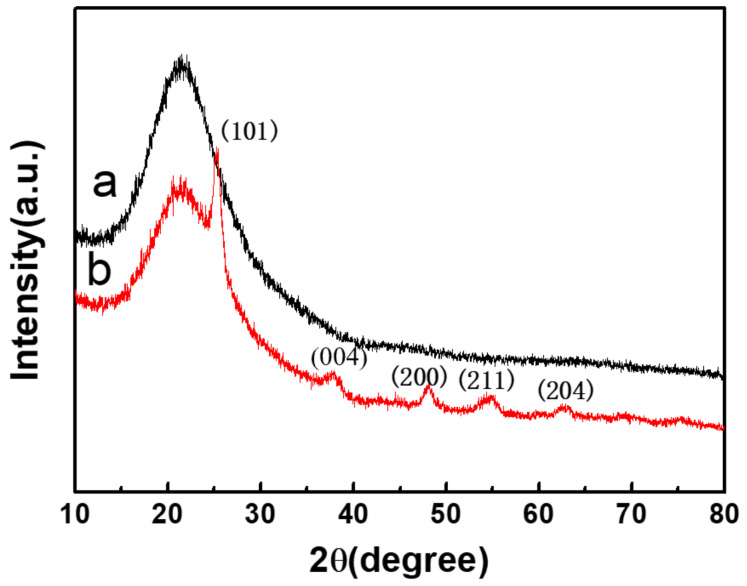
XRD patterns of SiO_2_ and TiO_2_-SiO_2_.

XRD patterns of SiO_2_ and TiO_2_-SiO_2_ were displayed in Figure 5. The hollow SiO_2_ microsphere was amorphous (curve a in Figure 5). The main peaks at 25.3°, 37.9°, 47.8°, 54.3°, and 63.0° in curve b could be assigned to the diffraction of the (101), (004), (200), (211), and (204) planes of anatase TiO_2_ [36]. Generally, the anatase TiO_2_ will change to rutile TiO_2_ after high-temperature roasting. However, no rutile formation was found in this sample because of the spatial grid effect after silicon addition, which improved the structural thermal stability of mesoporous TiO_2_ and inhibited the transformation of anatase to rutile [37].

**Figure 6:**
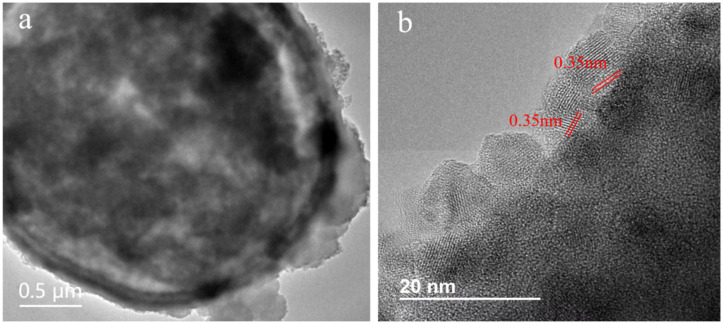
(**a**) TEM image and (**b**) high-resolution TEM image of the TiO_2_-SiO_2_.

To observe the microscopic morphology and internal structure of the prepared TiO_2_-SiO_2_, TEM characterization was carried out and the results were shown in Figure 6. In Figure 6a, a layer of material was successfully coated on the surface of SiO_2_. The spacing of the lattice plane in Figure 6b was 0.35 nm, which was consistent with the d value of the (101) plane of the anatase TiO_2_, confirming that the surfaces of the SiO_2_ were successfully coated by TiO_2_.

**Figure 7:**
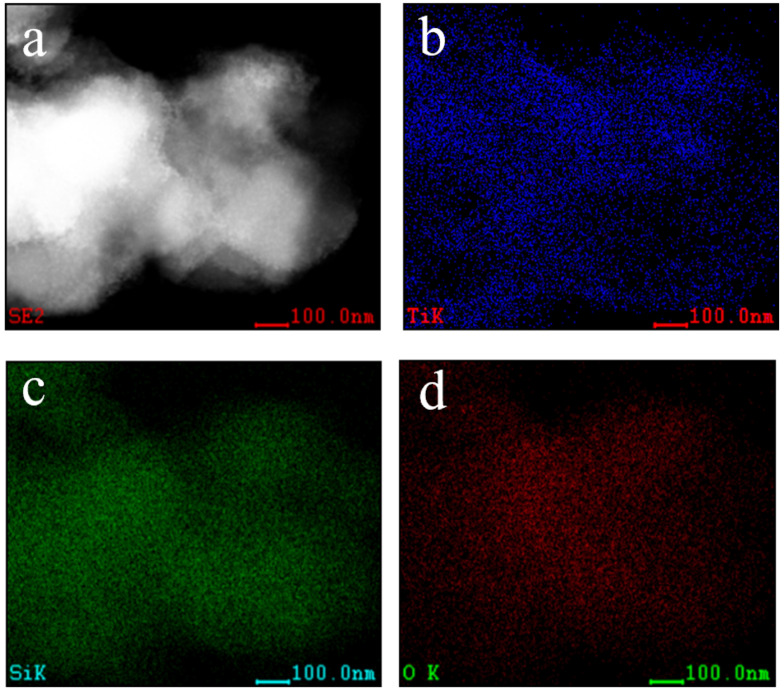
EDX elemental mapping investigation of (**a**) TiO_2_-SiO_2_ (**b**) Ti; (**c**) Si; (**d**) O.

In order to confirm the elemental composition and distribution of the TiO_2_-SiO_2_ catalyst, Si, O, and Ti elements were selected for an EDX surface scan. As shown in Figure 7, the distribution ranges of the O, Si, and Ti elements are consistent with the positions occupied by SiO_2_ and the distribution is very uniform. This reflects not only the O and Si element properties of SiO_2_, but also the fact that the titanium layer is successfully coated on the surface of the hollow SiO_2_ microspheres.

**Figure 8:**
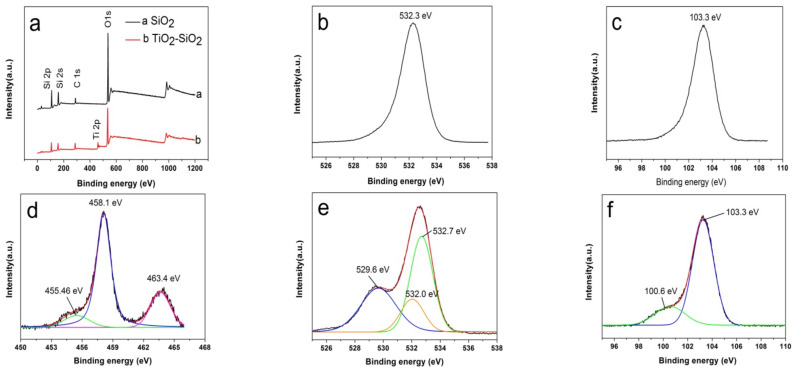
XPS spectra of SiO_2_ and TiO_2_-SiO_2_. (**a**) Survey scan; (**b**) O 1s of SiO_2_; (**c**) Si 2p of SiO_2_; (**d**) Ti 2p of TiO_2_-SiO_2_; (**e**) O 1s of TiO_2_-SiO_2_; (**f**) Si 2p of TiO_2_-SiO_2_.

The full XPS spectra of SiO_2_ and TiO_2_-SiO_2_ were shown in Figure 8a and several peaks corresponding to Si, C, Ti, and O elements could be observed. Figure 8b showed the XPS spectrum of O1s of the sample SiO_2_. The characteristic peak appeared at 532.3 eV and could be assigned to the binding energy of O 1s in Si-O-Si. The peak centered at around 103.3 eV in Figure 8c confirmed the presence of the Si element in SiO_2_. A Ti 2p XPS spectrum of TiO_2_-SiO_2_ in Figure 8d was fitted into three peaks. The peaks located at 458.1 eV and 463.4 eV were assigned to Ti 2p3/2 and Ti 2p1/2 of TiO_2_, respectively. A minor peak at 455.46 eV might be attributed to the low valence states of Ti [38]. The coating of TiO_2_ exerted a great influence on the O 1s XPS spectrum (Figure 8e), which could be split into three peaks. The peak located at 529.6 eV and 532.7 eV were related to Ti–O–Ti and Si–O–Si. The peak at 532.0 eV could be assigned to the binding energy of the Si-O-Ti species, indicating the bonding of TiO_2_ to SiO_2_ [39]. Figure 8f showed the XPS spectrum of Si 2p of the sample TiO_2_-SiO_2_. There were two characteristic peaks of Si 2p located at 100.6 eV and 103.3 eV, showing that the coating of TiO_2_ had an effect on the binding energy of the Si element.

The specific surface area (S_BET_), pore volume (V_P_), and pore diameter (D_P_) of different samples are summarized in Table 1. Compared with SiO_2_ without a template, the specific surface area, pore volume, and pore diameter of SiO_2_ produced by a yeast template increased by different degrees, and the increase in the specific surface area from 10.95 m^2^ g^−1^ to 15.97 m^2^ g^−1^ was mainly due to the successful formation of a hollow microsphere structure. When the hollow SiO_2_ microspheres were coated with titanium, the specific surface area, pore volume, and pore diameter increased, which may be beneficial by providing more active sites and increasing the catalytic activity of the catalyst.

**Figure 9:**
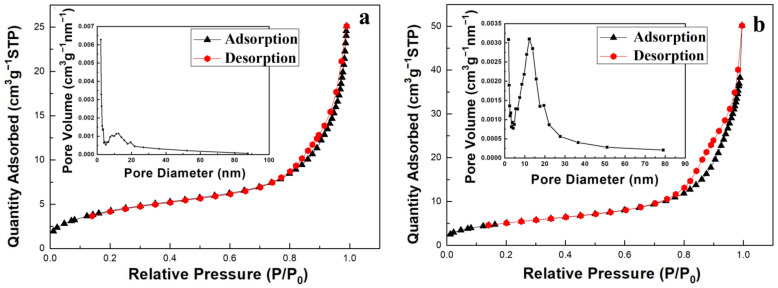
(**a**) N_2_ adsorption–desorption isotherm curves of SiO_2_. Inset is Barret Joyner Halenda (BJH) pore size distribution of SiO_2_; (**b**) N_2_ adsorption–desorption isotherm curves of TiO_2_-SiO_2_. Inset is BJH pore size distribution of TiO_2_- SiO_2_.

Figure 9a shows the N_2_ adsorption–desorption isothermal curve and Barret Joyner Halenda (BJH) pore diameter distribution of SiO_2_ prepared by the *P. pastoris* GS115 template. The N_2_ adsorption–desorption isothermal curve belongs to the Langmuir-type IV mesoporous channel adsorption curve. At P/Po = 0.8–1.0, small hysteresis rings appears, which maybe have been caused by slight changes in the pore diameter of SiO_2_ and the phenomenon of different pore sizes, and it could also have been caused by a small number of interstices between particles. The BJH pore diameter distribution diagram of SiO_2_ inserted in Figure 9a shows that the mesopore diameter is in the range of 5 to 18 nm.

Figure 9b shows the N_2_ adsorption–desorption isothermal curve and the BJH pore diameter distribution of TiO_2_-SiO_2_. At the low-pressure stage (P/Po < 0.8), there is a certain linear relationship between the adsorption amount and partial pressure, which may occur in a single layer of physical adsorption. When the partial pressure P/Po is approximately 0.8, the adsorption amount increases sharply and the adsorption enters the abrupt phase. The reason is that N_2_ condenses the capillary in the mesoporous channel. When the partial pressure P/Po continues to increase, another abrupt jump occurs and a hysteresis ring appears under high partial pressure. At this time, N_2_ condenses between material particles. It can be seen from the BJH pore diameter distribution curve inserted in Figure 9b that the sample pore diameter is mainly distributed between 5 and 20 nm. There are also concentrated holes, possibly caused by gaps between spherical particles of varying sizes.

### 3.3. Photocatalytic Activity

**Figure 10:**
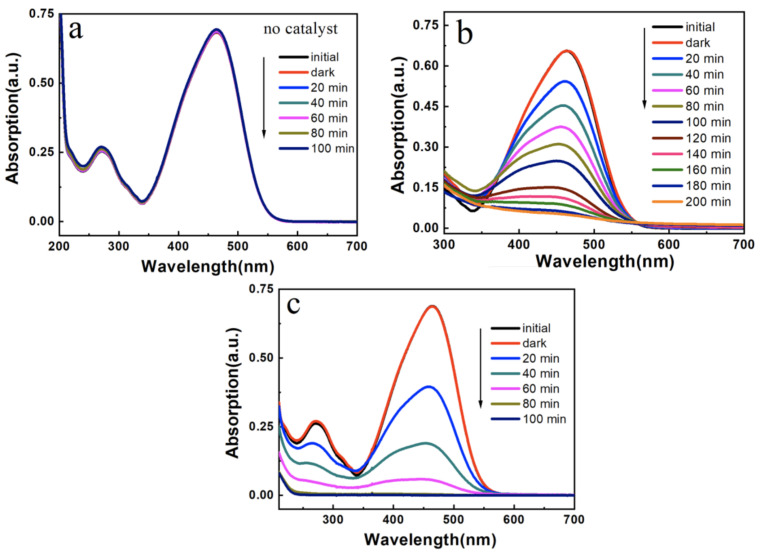
Photocatalytic activity of (**a**) no catalyst; (**b**) TiO_2_ prepared from *P. pastoris* GS115 as a template; (**c**) TiO_2_-SiO_2_ prepared from *P. pastoris* GS115 as a template for MO degradation.

**Figure A4:**
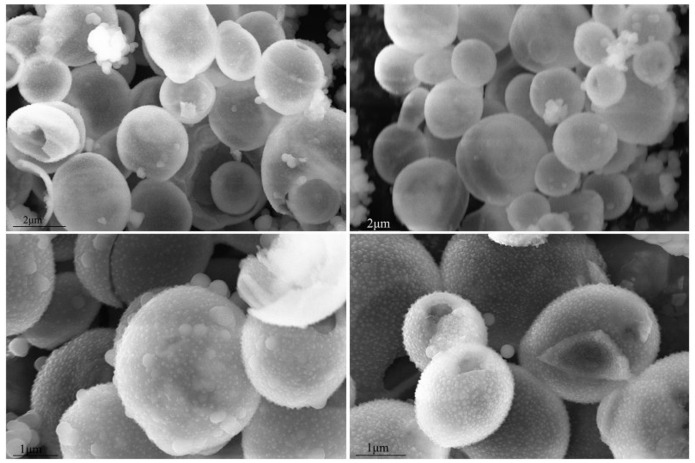
SEM images of TiO_2_ prepared from *P. pastoris* GS115 as a template.

It could be seen from the Figure 10a that the absorbance of the solution was basically unchanged when the catalyst was not added. After the catalyst was introduced, the absorbance of MO gradually decreased with the extension of time. Moreover, the hollow TiO_2_ microspheres were also prepared using *P. pastoris* GS115 as a template (see Figure A4). Comparing Figure 10b,c, it could be seen that the photocatalytic degradation ability of TiO_2_-SiO_2_ was higher than that of pure TiO_2_ prepared with *P. pastoris* GS115 as a template. The probable cause was that the introduction of SiO_2_ to TiO_2_ could reduce its surface energy to a certain extent and reduce its agglomeration, forming active hydroxyl radicals, and thereby enhancing the photocatalytic ability of TiO_2_ [40]. Jiang et al. [39] reported the preparation of hierarchical hollow TiO_2_@SiO_2_ composite microspherse and studied their photocatalytic performance on MO. The degradation rate was 99.7% after 3 h. Zhang et al. [41] prepared TiO_2_/SiO_2_ by the sol–gel method. The degradation rate of MO was 98.03% within 180 min using the 250 W mercury lamp as the light source. As shown in Figure 10c, the absorbance of MO was reduced to zero, indicating that MO was completely degraded in 100 min. The result suggests that the as-prepared TiO_2_-SiO_2_ microspheres exhibit an excellent photocatalytic activity.

**Figure 11:**
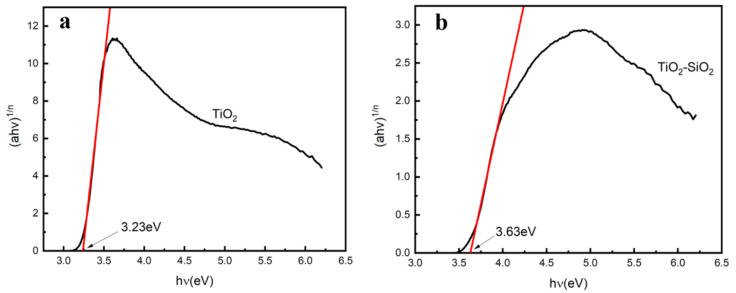
Estimated band gaps of (**a**) TiO_2_ prepared from *P. pastoris* GS115 as a template and (**b**) TiO_2_-SiO_2_ prepared from *P. pastoris* GS115 as a template based on the Tauc/Davis–Mott model.

The estimated band gaps of pure TiO_2_ and TiO_2_-SiO_2_ prepared with *P. pastoris* GS115 as a template are 3.23 eV and 3.63 eV, respectively (Figure 11). The band gap of TiO_2_-SiO_2_ is more than that of pure TiO_2_, indicating that the introduction of silicon leads to an increase in the band gap of the semiconductor and enhances the redox capacity of holes and electrons. Hence, the photocatalytic activity can be improved.

## 4. Conclusions

In summary, *P. pastoris* GS115 was employed as a typical microbe to demonstrate its potential in synthesizing high-efficient photocatalysts for the degradation of organic contaminants. Hollow SiO_2_ microspheres with a spherical morphology were successfully synthesized using the microbe template. The morphology and surface roughness of the hollow particles could be controlled by the reaction conditions. TiO_2_-SiO_2_ microspheres were successfully prepared by the hydrothermal process. Results indicated that TiO_2_-SiO_2_ kept in the favorable anatase phase of TiO_2_. The as-prepared TiO_2_-SiO_2_ exhibited good photocatalytic activity for the degradation of MO and the degradation rate could reach 99.9% in 100 min because of an increase in the band gap. This work is of great significance for employing microbes in the preparation of promising photocatalysts for large-scale practical application.

## Figures and Tables

**Table 1:** Specific surface area and pore size distribution parameters of different samples.

Sample	S_BET_ (m^2^g^−1^)	Vp (cm^3^g^−1^)	Dp (nm)
SiO_2_ without template	10.95	0.022	8.06
SiO_2_ with yeast template	15.97	0.039	9.73
TiO_2_-SiO_2_	18.88	0.071	13.01

## Data Availability

The data presented in this study are available on request from the corresponding author.

## Appendix A

When TEOS is added to a mixture of water and ethanol containing microorganism, TEOS is attached to the cell wall. After the addition of ammonia, TEOS begins to hydrolyze and the resulting SiO_2_ particles grow in the cell wall. Due to the slow hydrolysis rate of weak alkali in ammonia water, the adsorbed SiO_2_ nanoparticles on the cell wall surface have enough time to grow. After calcination, the template is removed and the hollow SiO_2_ microspheres are synthesized. TiO_2_-SiO_2_ microspheres are prepared by the hydrothermal process and the photocatalytic activity for the degradation of methyl orange (MO) at room temperature under Xe arc lamp acting as simulated sunlight was explored.

In order to investigate the influence of microbe templates on the preparation of SiO_2_, the samples were characterized by TG. Figure A2 shows the TG characterization diagrams of *Pichia pastoris* GS115 and uncalcined SiO_2_. From the TG spectrum of *P*. *pastoris* GS115, it can be seen that there is a small weight loss peak at 85 °C, which is mainly caused by the desorption of water on the surface of the sample; in the range of 200–700 °C, in the spectrum a distinct weight loss step appeared, which was caused by the gradual breakdown of the biomass molecules of the *P*. *pastoris* GS115. The TG spectrum of uncalcined SiO_2_ microspheres is similar to the weight loss peak of *P*. *pastoris* GS115, which indicates that some biomass may remain on the surface of the uncalcined SiO_2_ microspheres.

From the spectrum of uncalcined SiO_2_ in Figure A3, the structural water –OH anti scaling vibration peak appears at 3450 cm^−1^, and the peak near 1638cm^−1^ is the H–OH bend vibration peak of water. The peak at 955 cm^−1^ belongs to the bending vibration absorption peak of Si–OH, which is consistent with the literature reports. After calcination, the strong and wide absorption band at 1095 cm^−1^ is attributed to Si–O–Si anti scaling vibration peak, and the peak at 798cm^−1^ is attributed to Si–O symmetric stretching vibration peak.
